# Supplementary material for: Learning curve and surgical outcome of robotic assisted colorectal surgery with ERAS program
Source: Sci Rep. 2022 Nov 29;12:20566. doi: 10.1038/s41598-022-24665-w (PMC9709162; doi:10.1038/s41598-022-24665-w)
Supplement: Supplementary file 2 — Supplementary Information 2. [file 41598_2022_24665_MOESM2_ESM.zip › RAL Raw data-Ñ[▒K/126/HS_ñw╝╨░O▒Kñσ.pdf]

正式摘要(電子簽章：000000230460327)

W52 / W52 ☐ 英文標題(注意:無英文姓名-請補病人基本檔!) ☐【讀取】憑證

臺中榮民總醫院

出院病歷摘要

轉入號： 轉出號：

[redacted]

(8)流水編號	
(9)入院日期	110/11/17 (2021) 直腸外科 W52 - 143
(10)轉科日期	
(11)出院日期	110/11/22 (2021) 住院天數計 5 日

診斷

- 入院 : 1. Rectum cancer, 5cm FAV,
  - s/p Ufur+short course radiotherapy(4500cGy/25fr,5000cGy/25fr)
  2. HBV, under Beraclude
- 出院 : 1. Rectum cancer, 5cm FAV,
  - s/p Ufur+short course radiotherapy(4500cGy/25fr,5000cGy/25fr)
  - s/p Robotic assist LAR + loop ileostomy on 2021/11/18
  2. HBV, under Beraclude

主訴

- Bloody stool in 2021/06w

病史

- This is a 53 years old male patient denied underlying disease. According to the patient, he had bloody stool in 2021/06, then he went to Uuan Lin Christian H where colonoscopy was done, showed Anal ulcerative cancer, at 8-5 cm FAV, s/p partial polypectomy and biopsy. The pathology report confirm adenocarcinoma. Thus, he came to our CRS OPD for second opinion. Lab data showed CEA: 4.93, and HbsAg:1858. So he was refered to GI OPD first for HBV control. Then, the pateint received Ufur and Radiotherapy(5000cGy/25fr, 4500cGy/25fr). After finish neoadjuvant CCRT, this time he was admitted for scheduled operation.

體檢發現

-
  - General appearance :
    - easy looking , conscious clear , without cardiopulmonary distress
    - Vital signs自動帶入date 體溫(BT) 脈搏(P) 呼吸(R) 血壓(BP) 身高(BH) 體重(BW) BMI
      20211117 35.6℃ 75次/分 17次/分 148/84mmHg 166.9cm 66.1kg 23.7
    - Vital signs: B.P. 148/84 mmHg , P.R. 75 /min , R.R. 17 /min , B.T. 35.6 ℃
  - HEENT :
    - Eyes: no anemic conjunctiva, no icteric sclera, normal EOM
    - ENT: no otorrhea, no rhinorrhea, no erythematous mucosa, no injected tonsil, no deformity
  - Neck: supple, freely movable, trachea midline,no deformity, no engorged jugular vein , no palpable thyroid or lymph node enlargement
  - Chest:
    - Inspection: Grossly normal, symmetric and fully expansion, no accessory muscle used, no operation scar, no spider angiomata, no gynecomastia
    - Palpation: no palpable mass or nodule, no subcutaneous emphysema, no tenderness
    - Ausculation: clear breathing sound
  - Heart:
    - Palpation: no thrill, no heave
    - Percussion: normal heart size
    - Ausculation: regular heart beat, normal S1, S2, no gallop, no murmur
  - Extremities: No deformity, full ranged of motion without limitation, no clubbing finger or nail cyanosis, no pitting edema, no ulcer or pigmentation, no obvious skin lesion, no varicose vein, no bone, joint or muscle tenderness

- **CRS CONDITION**
  - Karnofsky performance status: (100) Normal, no complaints, no evidence of disease
  - PE of Abdomen:
  - CEA: 3.24 ng/ml
  - Imaging Studies:
  - Colonoscope report: .
  - Figures:
    1. colon & rectum

手術日期及方法

- 手術日期及方法20211118 1. Robotic (XI) assisted TME+ loop ileostomy
  2. Intraoperative colonoscopy : (-).
  3. High ligation (+) : At IMA root (Via robotic clips). IMV was ligated via endoclip.
  4. Take down from the splenic flexure : (-)
  5. Proximal cut end: > 10 cm, distal cut end: 1.5 cm
  6. No drainage tube was inserted
  7. The residual proximal D colon was sutured to distal rectal stump via hand sewn with 1-0 Vicryl
  8. Loop ileostomy (About 15~20cm from IC valve) was done over RLQ of abdomen

住院治療經過

- After admission, he received Robotic assist LAR + loop ileostomy on 2021/11/18. Stool passage was noticed on 11/19 and he start try bland I diet. He start try bland II diet on 11/20 and remove Foley catheter. He try bland III well and removed CVC on 11/22, then discharged. OPD f/u

合併症

一般檢查

- 累積報告 :

DATE	TIME	ALB	TP	BIL.T	BIL.D	ALKP	AST	ALT	LDH	AMY	LIPASE	NH3	Ferritin	UA
1101117	1700	4.6	6.9	1.0	0.2		24	23						5.1
1101118	1642			1.36			18	19						

DATE	TIME	WBC	RBC	HGB	HCT	MCV	PLT	BLST	NEUT	BAND	SEG	LYM	MON	EOS	BAS
1101117	1657	4470	3.72	14.1	40.3	108.3	165		73.6			15.9	9.2	0.9	0.4
1101118	1637	17840		13.6	37.6		158		94.1			3.2	2.3	0.2	0.2

DATE	TIME	NA	K	CL	BUN	CREAT	eGFR	CA	Mg	P	CRP	ESR	PCT	D-Dimer	LACTATE
1101117	1700	140	4.3		19	0.84	101.59	8.8			0.022				
1101118	1642	138	3.6		13	0.75	115.79	8.5							19.2

DATE	TIME	GLUCOSE	HbA1C	CHOL	TG	HDL	LDL	KETONE	CK	CKMB	TRO-T	PT-P	APTT-P	NT-pro BNP
1101117	1658											11.8	25.0	
1101117	1659	97												
1101117	1700			170	43	82	82							
1101117	1701		5.2											

特殊檢查

病理報告

其它

出院時情況

- 3.改門診治療

出院指示

藥名	劑量	劑量單位	用法	頻次	數量	數量單位	現況	天數	單位
(口)Takepron OD tab 30mg	1	TAB	PO	QDAC	8	TAB	TH	8	天
Ultracet tab	1	TAB	PO	Q6H	32	TAB	TH	8	天
Silence tab 1mg	1	TAB	PO	HSPRN	8	TAB	TH	8	天
OCAS Harnalidge 0.4 mg	1	TAB	PO	QDAC	8	TAB	TH	8	天

出院計劃

- 門診日期
  - date on 2021/11/30

主治醫師 2062F 蔣鋒帆 / 住院醫師 2339C 蔡濬樟

正式摘要(電子簽章：**000000230460327)**

W52 / W52

doctorRank:B
